# Multivariate and Multiscale Data Assimilation in Terrestrial Systems: A Review

**DOI:** 10.3390/s121216291

**Published:** 2012-11-26

**Authors:** Carsten Montzka, Valentijn R. N. Pauwels, Harrie-Jan Hendricks Franssen, Xujun Han, Harry Vereecken

**Affiliations:** 1 Forschungszentrum Jülich GmbH, Institute of Bio- and Geosciences: Agrosphere (IBG 3), Jülich 52425, Germany; E-Mails: h.hendricks-franssen@fz-juelich.de (H.-J.H.F.); h.vereecken@fz-juelich.de (H.V.); 2 Department of Civil Engineering, Monash University, Clayton, Victoria 3800, Australia; E-Mail: Valentijn.Pauwels@monash.edu; 3 Cold and Arid Regions Environment Engineering Research Institute (CAREERI), CAS, Lanzhou 730000, China; E-Mail: hanxj@lzb.ac.cn

**Keywords:** data assimilation, multiscale, multivariate, modeling, Ensemble Kalman Filter, Particle Filter, variational methods

## Abstract

More and more terrestrial observational networks are being established to monitor climatic, hydrological and land-use changes in different regions of the World. In these networks, time series of states and fluxes are recorded in an automated manner, often with a high temporal resolution. These data are important for the understanding of water, energy, and/or matter fluxes, as well as their biological and physical drivers and interactions with and within the terrestrial system. Similarly, the number and accuracy of variables, which can be observed by spaceborne sensors, are increasing. Data assimilation (DA) methods utilize these observations in terrestrial models in order to increase process knowledge as well as to improve forecasts for the system being studied. The widely implemented automation in observing environmental states and fluxes makes an operational computation more and more feasible, and it opens the perspective of short-time forecasts of the state of terrestrial systems. In this paper, we review the state of the art with respect to DA focusing on the joint assimilation of observational data precedents from different spatial scales and different data types. An introduction is given to different DA methods, such as the Ensemble Kalman Filter (EnKF), Particle Filter (PF) and variational methods (3/4D-VAR). In this review, we distinguish between four major DA approaches: (1) univariate single-scale DA (UVSS), which is the approach used in the majority of published DA applications, (2) univariate multiscale DA (UVMS) referring to a methodology which acknowledges that at least some of the assimilated data are measured at a different scale than the computational grid scale, (3) multivariate single-scale DA (MVSS) dealing with the assimilation of at least two different data types, and (4) combined multivariate multiscale DA (MVMS). Finally, we conclude with a discussion on the advantages and disadvantages of the assimilation of multiple data types in a simulation model. Existing approaches can be used to simultaneously update several model states and model parameters if applicable. In other words, the basic principles for multivariate data assimilation are already available. We argue that a better understanding of the measurement errors for different observation types, improved estimates of observation bias and improved multiscale assimilation methods for data which scale nonlinearly is important to properly weight them in multiscale multivariate data assimilation. In this context, improved cross-validation of different data types, and increased ground truth verification of remote sensing products are required.

## 1. Introduction

The basic idea behind data assimilation (DA) is to combine complementary information from measurements and models of the Earth system and thus optimally estimate geophysical fields of interest [1]. It allows model simulations to be updated with observation data, for example in the carbon cycle [2], plant phenology [3] or hydrologic remote sensing [4]. The theory of DA in the Earth sciences rests on the mathematical framework of estimation theory [1,5]. More advanced DA methods also provide a framework for incorporating model errors and for quantifying prediction uncertainties [6] or updating model parameters [7].

In the context of climate change and land-use change, more and more terrestrial observational networks are being established to monitor states and fluxes in an effort to understand water, energy, or matter fluxes, as well as their biological and physical drivers and interactions with and within the terrestrial system. Examples of these networks include the global FLUXNET [8], the US Soil Climate Analysis Network (SCAN) [9], the US Snowpack Telemetry Network (SNOTEL) [10], the European Integrated Carbon Observation System (ICOS), and the German Terrestrial Environmental Observatories (TERENO) [11]. Within these networks, a huge amount of data from different sensors is recorded on different temporal and spatial scales. Moreover, a large number of Earth observation satellites have been launched, and products are being delivered for use in terrestrial models. Examples are the leaf area index (LAI), the fraction of absorbed photosynthetic active radiation (FPAR) and the land surface temperature (LST) retrieved by the Moderate Resolution Imaging Spectroradiometer (MODIS) [12,13], the soil moisture retrieved by the Soil Moisture and Ocean Salinity (SMOS) Mission [14], and the snow water equivalent as retrieved by the Advanced Microwave Scanning Radiometer-EOS (AMSR-E) [15]. These global satellite products are generated in an operational manner, *i.e.*, consistent time series of the variables mentioned are available.

The potential of these multiple data sets as well as their combination is often not fully exploited. DA, which is defined as the updating of modeled state variables (and possibly also other model components like parameters and forcings) using externally obtained data sets, has been applied in the Earth sciences for decades. DA techniques, such as the Ensemble Kalman Filter [16], the Particle Filter [17,18] or variational methods like 4D-VAR [19], integrate observations into terrestrial models for an enhanced description of real environmental conditions. Numerous applications of single-state assimilation have already been published, e.g., in the context of the hydrological cycle [20–30], the energy balance [31], plant physiology [32–34], the carbon cycle [35,36], nutrient cycles [37] and zoology [38]. However, the additional information contained in data from multiple-state variables compared to single-state variables may significantly improve the description of the full system by assimilation. In this context, existing DA techniques can be modified accordingly or new approaches can be developed to improve performance.

The objective of this paper is to review the state of the art of multivariate and multiscale DA techniques in terrestrial systems, to detect current limitations for the use of multivariate and multiscale DA and to provide guidance for further methodological developments and potential areas of application.

## 2. Data Assimilation Theory

**Figure 1.**
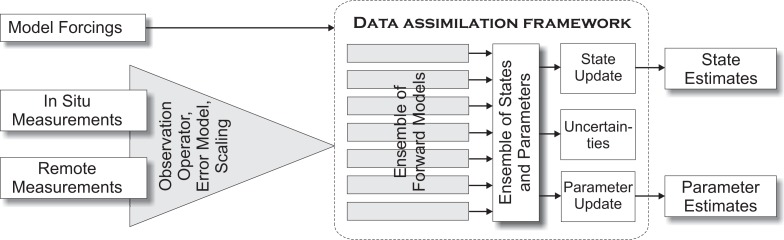
Ensemble-based DA system. Measurements are integrated into a DA framework by an observation operator for comparison with ensemble states for state (and parameter) updates. The scheme is presented for one time step only, the sequential character of DA is generated by new model forcings and new measurements initiating a new ensemble of forward models for the next time step.

In this section, we present a brief introduction to three prominent DA techniques, namely the Ensemble Kalman Filter (EnKF), the Particle Filter (PF) and variational methods (VAR). EnKF and PF are Bayesian-based approaches, whereas VAR uses the minimization of a cost function [in principle, EnKF can also be derived from a cost function minimization under the hypotheses of a linear model and Gaussian probability density functions (pdfs)]. The algorithms, or their derivatives, are widely used in environmental modeling. The general principle of operation will be clarified, which is important for the understanding of multivariate and multiscale DA techniques. The methods discussed here are well suited for parallel computation, since they make use of ensemble members. This is also true for VAR methods when applied in an ensemble approach [39]. Each of the ensemble members corresponds to a model run for which a separate CPU processor can be used. Figure 1 presents a schematic overview of ensemble-based DA methods. Measurements are integrated into a DA framework by an observation operator for comparison with ensemble states for state update, and in some cases for parameter update. The results are enhanced state and parameter estimates which include their uncertainties.

DA algorithms based on recursive Bayesian estimation techniques first emerged with the Kalman filter [40]. Based on this theory, the Extended Kalman Filter (EKF) was then derived for the optimization of nonlinear systems and Hoeben and Troch [41] provided an overview of this methodology. The major drawback in the application of the EKF is the need to linearize the model equations. In order to overcome this problem, the EnKF was developed [16]. The Ensemble Kalman Filter (EnKF), a Monte Carlo implementation of Bayesian updating, proposed by Evensen [16] and clarified by Burgers *et al*. [42], is widely used in environmental applications. It reduces the computational demand relative to the EKF by integrating an ensemble of states from which the covariances are obtained at each update. It thereby avoids the need to linearize the model equations for the propagation of the error covariance [43,44]. Similar to the Kalman filter, the EnKF relies on a Gaussian assumption of model and observation errors, which may not be valid in environmental modeling [45–47]. In addition, linear updating of model states using this method reduces its applicability for highly nonlinear systems [7]. Another albeit more CPU-intensive alternative is the use of sequential Monte Carlo methods in the form of PF [18,48]. The PF differs from classical Kalman Filtering methods as it can handle the propagation of non-Gaussian distributions through nonlinear models. Both PF and EnKF are Monte Carlo techniques which use samples (*i.e.*, ensemble members or particles) to estimate the underlying pdf of model states and parameters. Comparative studies of both EnKF and PF can be found, e.g., in Weerts *et al*. [49], Han and Li [45], Jardak *et al*. [50], Pasetto *et al*. [51], Leisenring and Moradkhani [52], and DeChant and Moradkhani [53].

Variational DA (VAR) is a very successful technique for operational numerical weather prediction because it can be efficiently used in realistic, complex systems. It was introduced in a three-dimensional form (3D-VAR) by Parrish and Derber [54], and then applied in a four-dimensional form (4D-VAR) using an adjoint model to include the time dimension [55]. However, the variational method itself does not provide any estimate of predictive uncertainty. The adjoint method calculates exact gradient information of the objective function that is to be optimized. Moreover, compared with EnKF, the advantage of 3/4D-VAR is the fact that nonlinear dependencies between observations and state variables can be taken into account without any approximation.

### 2.1. Ensemble Kalman Filter (EnKF)

In the application of the EnKF, the system state at time step k − 1 (x_k−1_) is propagated to time step k as follows:

(1) $${\text{x}}_{\text{k}}={\text{f}}_{\text{k},\text{k}-1}({\text{x}}_{\text{k}-1},\;{\text{w}}_{\text{k}-1})$$

f_k,k−1_(.) is a nonlinear operator representing the model in state space, including the model parameters and the meteorological forcings. w_k−1_ is the process noise. This is a white-noise term with zero mean and covariance matrix Q_k−1_, and it summarizes all the uncertainties caused by the model formulation, the forcing data, and the model parameters. The system is observed as follows:

(2) $${\text{y}}_{\text{k}}={\text{h}}_{\text{k}}({\text{x}}_{\text{k}},\;{\text{v}}_{\text{k}})$$

h_k_(.) is a nonlinear function, relating the state variables to the observations. v_k_ is the observation noise, which is a white-noise term with zero mean and covariance matrix R_k_. It should be noted that for all time steps w_k_ and v_k_ are independent.

Instead of propagating one single model realization, the EnKF propagates an ensemble of model realizations. The spread in the ensemble at each time step is an estimate of the uncertainty in the model results. The a priori (before the update) state variables of a single ensemble member i are stored in the vector ${\text{x}}_{\text{k}}^{\text{i}-}$. The superscript .^−^ indicates an a priori estimate. This vector is obtained by propagating each ensemble member i:

(3) $${\hat{\text{x}}}_{\text{k}}^{\text{i}-}={\text{f}}_{\text{k},\text{k}-1}\left({\hat{\text{x}}}_{\text{k}-1}^{\text{i}+},\;{\text{w}}_{\text{k}-1}^{\text{i}}\right)$$

The apex $\hat{.}$ indicates the ensemble mean and the superscript .^+^ indicates an a posteriori estimate (after the update). ${\text{w}}_{\text{k}-1}^{\text{i}}$ is a realization of the model error, obtained by a perturbation of the model parameters and meteorological forcings. The background error covariance ${\text{P}}_{\text{k}}^{-}$ is then calculated to estimate the forecast uncertainty:

(4) $$\{\begin{array}{ccc} {\text{P}}_{\text{k}}^{-} & = & \frac{1}{\text{N}-1}{\text{D}}_{\text{k}}\;{\text{D}}_{\text{k}}^{\text{T}}\\ {\text{D}}_{\text{k}} & = & [{\text{x}}_{\text{k}}^{1-}-{\text{x}}_{\text{k}}^{-},...,{\text{x}}_{\text{k}}^{\text{N}-}-{\text{x}}_{\text{k}}^{-}\\ {\text{x}}_{\text{k}}^{-} & = & \frac{1}{\text{N}}\sum_{\text{i}=1}^{\text{N}}{\text{x}}_{\text{k},\text{i}}^{\text{i}-} \end{array}$$

N is the number of ensemble members and the superscript .^T^ indicates the transpose operator. The Kalman gain K_K_ is then calculated as:

(5) $${\text{K}}_{\text{k}}={\text{P}}_{\text{k}}^{-}{\text{H}}_{\text{k}}^{\text{T}}{\left[{\text{H}}_{\text{k}}{\text{P}}_{\text{k}}^{-}{\text{H}}_{\text{k}}^{\text{T}}+{\text{H}}_{\text{k}}\right]}^{-1}$$

where H_k_ is the Jacobian of the observation system [Equation (2)]. If a proxy of the modeled state variables is used to update the system (for example, if radar backscatter values are used to update the modeled soil moisture profile), the observation system thus needs to be linearized in order to calculate H_k_. In order to bypass the need to linearize the observation system, ${\text{P}}_{\text{k}}^{-}{\text{H}}_{\text{k}}^{\text{T}}$ can be calculated as the covariance between the state and the measurement predictions, and ${\text{H}}_{\text{k}}{\text{P}}_{\text{k}}^{-}{\text{H}}_{\text{k}}^{\text{T}}$ as the covariance of the measurement predictions [in a similar way to Equation (4)]. Pauwels and De Lannoy [56] have proven that the use of the ensemble results outperforms the linearization of the observing system and the use of H_k_ for the calculation of the Kalman gain. If N is larger than the number of observations, the rank of the matrix that needs to be inverted is always the same as the number of observations that are used to update the system. Using the Kalman gain, the states of the individual ensemble members are then updated:

(6) $${\text{x}}_{\text{k}}^{\text{i}+}={\text{x}}_{\text{k}}^{\text{i}-}+{\text{K}}_{\text{k}}[{\text{y}}_{\text{k}}-{\text{h}}_{\text{k}}({\text{x}}_{\text{k}}^{\text{i}-},\;{\text{v}}_{\text{k}}^{\text{i}})]$$

where ${\text{x}}_{\text{k}}^{\text{i}+}$ is the *a posteriori* vector (after the update), and ${\text{v}}_{\text{k}}^{\text{i}}$ is a realization of the observation error. ${\text{x}}_{\text{k}}^{\text{i}+}$ can then be propagated to the next time step.

The EnKF is a sophisticated sequential DA method [57], which can be easily operationally implemented in forecast models to make use of new observations from different kinds of measurement systems. In contrast to the classical Kalman filter, the EnKF uses the sample covariance instead of the theoretical covariance matrix. Several realizations of model predictions with perturbed initial conditions, forcings and/or parameters are calculated until new observations become available. Only the states at the current time step are updated in the traditional formulation of EnKF. The updating of the states is based on the prediction with the (often nonlinear) simulation model, a measurement model and an updating scheme based on the recursive application of Bayes’ rule. The EnKF assumes that all probability density functions involved in the updating step are Gaussian. The original formulation of the EnKF by Evensen [16] was modified slightly for randomly perturbing measurements in order to take measurement error into account [42,58].

A variant of the Ensemble Kalman Filter is the Ensemble Kalman Smoother (EnKS) [59]. For EnKS, Equation (4) is modified so that the background error covariance matrix not only includes covariances among states for time step k, but also covariances between states at time step k and former time steps k − 1, k − 2, … . As a consequence, the Kalman gain [Equation (5)] is also modified, and spreads updates related to deviations between model predictions and measurements [Equation (6)] not only to the rest of the model states at time step k, but also to the model states of former time steps k − 1, k − 2, … .The EnKS is often applied to update the model states of a limited time window before time step k, and not the complete model history. Updating the complete model history demands large amounts of RAM on computers, and is less important if the correlation between a certain time step in the past and the current time step k is very weak. Both EnKF and EnKS can be extended to update both states and parameters, using for example an augmented state-vector approach. In this case, parameter values are sequentially improved profiting from correlations between states and parameters. In theory, EnKF and EnKS should give the same parameter estimates and the same state estimates for the last time step k. However, it is expected that EnKS will provide better state estimates for past time steps k − 1, k − 2, … [57].

A further variant related to EnKF and EnKS is the Ensemble Smoother (ES) [60]. In this case, all observations are assimilated simultaneously, over a given model simulation period. The deviations between the ensemble model simulations and measurements at the different time steps are used to update all past model states and parameters. In this case, the background error covariance matrix contains covariances between all model states for all time steps, and between the model states (at all time steps) and parameters. This method is in fact an inverse modeling method, and normally focuses on parameter estimation. EnKF was shown to outperform ES according to previous studies [60,61].

EnKF became popular in several fields of research for updating model states with aid of the sequential assimilation of measurements. Examples of remote sensing DA include the updating of soil moisture contents [43,62–64], snow water equivalent [65], runoff [66], groundwater storage [67] and vegetation characteristics in agroecosystems [68]. *In situ* measurements were assimilated in order to update, e.g., streamflow [69] or reservoir characteristics [70]. In DA with EnKF, seismic data has also been used by Skjervheim *et al*. [71] and synthetic electrical resistivity data were used to update groundwater states and parameters by Camporese *et al*. [72]. Recently, EnKF has been used to update both states and parameters [7,73–75], which is of special interest if parameters are time-dependent like riverbed hydraulic conductivities [76]. The EnKF does not ensure the consistency of the updated states or model parameters with physical constraints, e.g., the mass balance. In order to solve this problem, Wen and Chen [77,78] introduced a confirmation step to ensure consistency. This approach was extended by Gu and Oliver [79] with an iterative EnKF, where the iterations check the validity of physical constraints. The EnKS has been applied less frequently to update model states. An example is updating current and past soil moisture contents with remote sensing information [80,81]. Bateni and Entekhabi [82] also estimated model parameters with EnKS (evaporative fraction and a bulk scalar transfer coefficient), combining a land surface model and remotely sensed land surface temperature.

### 2.2. Particle Filter (PF)

Particle filters [18] are the sequential or online analogue of Markov Chain Monte Carlo (MCMC) batch methods. With sufficient samples, the so-called particles approach the Bayesian optimal estimate. Particle filters share the same forecast step as EnKF. The EnKF is characterized as a special case of the PF, where the Bayesian update step is approximated with a linear update step in the EnKF using only the two first moments of the predicted probability density function (pdf) [73]. A PF does not have this limitation during the update step and manages the propagation of non-Gaussian distribution more flexibly through nonlinear models [48].

The PF uses the same system description as the EnKF (Equation (1) and Equation (2)), and has been introduced in hydrology by Moradkhani *et al*. [48]. Plaza *et al*. [83] provided a detailed explanation of the algorithm. Only a short description will be provided here. In recursive Bayesian filtering, the solution to the estimation problem consists of two steps: the prediction and correction steps. These steps are formulated as follows:

(7) $$\text{p}({\text{x}}_{\text{k}}|{\text{y}}_{1:\text{k}-1})=\int \text{p}({\text{x}}_{\text{k}}|{\text{x}}_{\text{k}-1})\text{p}({\text{x}}_{\text{k}-1}|{\text{y}}_{1:\text{k}-1}){\text{dx}}_{\text{t}-1}$$

(8) $$\text{p}({\text{x}}_{\text{k}}|{\text{y}}_{1:\text{k}})=\frac{\text{p}({\text{y}}_{\text{k}}|{\text{x}}_{\text{k}})\text{p}({\text{x}}_{\text{k}}|{\text{y}}_{1:\text{k}-1})}{\int \text{p}({\text{y}}_{\text{k}}|{\text{x}}_{\text{k}})\text{p}({\text{x}}_{\text{k}}|{\text{y}}_{1:\text{k}-1}){\text{dx}}_{\text{k}}}$$

In the prediction step [Equation (7)], the posterior pdf p(x_k_|y_1:k−1_) is obtained based on the fact that the transition pdf p(x_k_|x_k−1_) and the prior pdf at time step k − 1 are known, whereas in the correction step [Equation (8)], the prior pdf is corrected using the information from the likelihood pdf p(y_k_|x_k_), and the posterior pdf p(x_k_|y_1:k_) is derived. The analytical solution to Equations (7) and (8) is difficult to determine since the evaluation of the integrals might be intractable. Particle filters are a set of algorithms which approximate the posterior pdf by a group of random samples. In more detail, the integrals are mapped to discrete sums:

(9) $$\text{p}({\text{x}}_{\text{k}}|{\text{y}}_{1:\text{k}})\approx \hat{\text{p}}({\text{x}}_{\text{k}}|{\text{y}}_{1:\text{k}})=\frac{1}{\text{N}}\sum_{\text{i}=1}^{\text{N}}\delta ({\text{x}}_{\text{k}}-{\text{x}}_{\text{k}}^{\text{i}})$$

where N is the number of particles. The particles $\left\{{\text{x}}_{\text{k}}^{\text{i}};\text{i}=1...\text{N}\right\}$ should be sampled from the posterior pdf and δ is the Dirac measure. The Dirac measure is given by:

(10) $$\delta_{\text{x}}(\text{X})=\{\begin{array}{ll} 0 & \text{if x}∋\text{X},\\ 1 & \text{if x}\in \text{X}. \end{array}$$

where x is a possible element of set X. At this point, drawing particles is unfeasible since the posterior pdf is unknown. Nevertheless, it is viable to draw particles from a known proposal pdf (also called importance pdf). This is the basis of the importance sampling principle. Sequential Importance Sampling (SIS) is the recursive version of the importance sampling MC method and the particle filters are based on the SIS approach. This approach approximates the posterior pdf by a set of weighted particles as follows:

(11) $$\hat{\text{p}}({\text{x}}_{\text{k}}|{\text{y}}_{1:\text{k}})=\sum_{\text{i}=1}^{\text{N}}{\hat{\text{w}}}_{\text{k}}^{\text{i}}\delta ({\text{x}}_{\text{k}}-{\text{x}}_{\text{k}}^{\text{i}})$$

where ${\hat{\text{w}}}_{\text{k}}^{\text{i}}$ are the normalized importance weights associated with the particles which are drawn from the proposal pdf. Considering that the system state evolves according to a Markov process, and applying recursion to the filtering problem, the recursive expression for the importance weights is given by:

(12) $${\text{w}}_{\text{k}}^{\text{i}}={\text{w}}_{\text{k}-1}^{\text{i}}\frac{\text{p}({\text{y}}_{\text{k}}|{\text{x}}_{\text{k}}^{\text{i}})\text{p}({\text{x}}_{\text{k}}^{\text{i}}|{\text{x}}_{\text{k}-1}^{\text{i}})}{\text{q}({\text{x}}_{\text{k}}^{\text{i}}|{\text{x}}_{0:\text{k}-1}^{\text{i}},y_{1:\text{k}})}$$

The selection of the proposal pdf $\text{q}(.|{\text{x}}_{0:\text{k}-1}^{\text{i}},{\text{y}}_{1:\text{k}})$ is extremely important in the design stage of the SIS filter. The filter performance mainly depends on how well the proposal pdf approximates the posterior pdf. In Doucet *et al*. [84], an optimal choice for the importance density function is proposed:

(13) $$\text{q}({\text{x}}_{\text{k}}^{\text{i}}|{\text{x}}_{0:\text{k}-1}^{\text{i}},y_{1:\text{k}})=\text{p}({\text{x}}_{\text{k}}|{\text{x}}_{0:\text{k}-1},y_{1:\text{k}})$$

This pdf is optimal in the sense that it minimizes the variance of the importance weights. However, the application of Equation (13) is complex from the implementation point of view. A common choice of the proposal is the transition prior function [18,85]:

(14) $$\text{q}({\text{x}}_{\text{k}}^{\text{i}}|{\text{x}}_{0:\text{k}-1}^{\text{i}},\;y_{1:\text{k}})=\text{p}({\text{x}}_{\text{k}}^{\text{i}}|{\text{x}}_{0:\text{k}-1}^{\text{i}})$$

A drawback of this approach is the lack of information regarding the model errors in the computation of the importance weights. This limitation can affect the performance of the particle filter. The choice of the transition prior to the proposal simplifies Equation (12) resulting in an expression where the importance weights depend on their past values and also on the likelihood pdf. The normalized weights are given by:

(15) $${\tilde{\text{w}}}_{\text{k}}^{\text{i}}=\frac{{\text{w}}_{\text{k}}^{\text{i}}}{\sum_{\text{i}=1}^{\text{N}}{\text{w}}_{\text{k}}^{\text{i}}}$$

**Figure 2.**
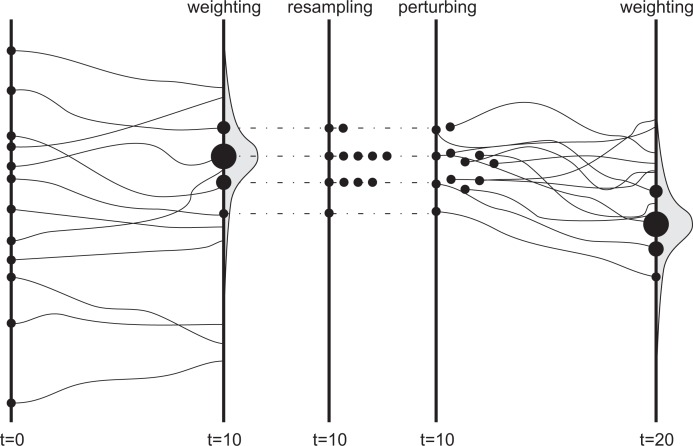
The importance resampling particle filter with 12 particles (modified according to van Leeuwen [86]). The model variable runs along the vertical axis, the weight of each particle corresponds to the size of the circles on this axis. t = 0, t = 10 and t = 20 denotes time, with observations at a time interval of 10 time units. All particles have equal weight at time zero. At time 10, the particles are weighted according to likelihood and resampled to obtain an equal-weight ensemble. Some studies perform a perturbation of states and/or parameters after resampling in order to avoid sample impoverishment.

The denominator in Equation (15) normalizes the weights. After several updates, a few particles may remain with high weights and a large fraction may have a weight of zero. To avoid this degeneracy of particles, a resampling step is performed. Particles with low weights are more likely to be substituted by replicates of particles with high weights, where the probability of a selection is equal to the individual weight. After resampling, the particles are equally weighted. This method is also presented in Figure 2 modified according to van Leeuwen [86]. Finally, the best estimate of the states consists of the weighted means for these states for the particle set $\left\{{\hat{\text{x}}}_{\text{k}}^{\text{i}-},\;{\tilde{\text{w}}}_{\text{k}}^{\text{i}}\right\}$. The SIS filter poses the problem of particle depletion, this problem is caused by increased variance over time as stated in Kong *et al*. [87] and Doucet *et al*. [84]. Methods to overcome this problem are Sequential Resampling [18] and Residual Resampling [88].

As shown in Figure 2, the particle replicates have the same values as the particle that was duplicated, which is ineffective as it might give the same model predictions (if model forcings are deterministic). Therefore, if parameters are stochastic and are a dominant source of uncertainty, Moradkhani *et al*. [48] recommend a minor parameter particle perturbation after each assimilation step in order to avoid sample impoverishment.

The resampling step is an essential part of the PF methodology and is necessary to improve the efficiency of PF. Often the Sampling Importance Resampling PF is used (SIR-PF) [18]. Alternative methods are Residual Sampling [88] and Stochastic Universal Resampling [85]. The latter have the advantage that they reduce sampling noise, and it has been shown that stochastic universal sampling has the lowest sampling noise [85]. Resampling slows down the degeneration of the weights, but it does not solve the problem. Resampling using Markov Chain Monte Carlo (MCMC) techniques is an interesting alternative, which needs to be exploited further because it could avoid the degeneration of weights. However, such resampling would require additional iterations, which are likely to cost a lot of additional CPU time [89]. More detailed discussions of the particle filter with different resampling strategies can be found, e.g., in [1,4,45,48,86].

PF has been applied for parameter estimation in rainfall-runoff modeling [48], to estimate groundwater recharge [90], to improve land surface states as well as water and energy fluxes [91], to improve surface soil moisture and model parameters [92], and to improve infiltration in a full-scale model of a dyke [29].

In the examples mentioned above, the models used had a limited number of states and only a few parameters were estimated. This is the main limitation of the PF. A very large number of particles is needed for adequate sampling of a high-dimensional state space. Resampling only partially alleviates this fundamental problem, and it is unclear whether the introduction of MCMC in the context of the PF could be CPU-efficient in the future. Neither improved estimates of the proposal distribution with Gaussian approximations nor the use of future measurement data yielded a breakthrough in this respect. Therefore, although the PF is one of the important alternatives to the EnKF, it currently needs an excessive amount of CPU time and its implementation in combination with large simulation models is not feasible. Application in high-performance computing and on parallelized architectures may help to overcome this problem. Whereas drawing samples in the state space and computing proper importance weights of each sample can be performed in parallel using a separate node for each particle, standard resampling techniques require strong interaction between nodes. However, resampling techniques specifically designed for parallel computation have recently been proposed [93].

### 2.3. Variational Assimilation (VAR)

In variational DA, the state vector x_k−n_ is calculated, which minimizes a cost function. This cost function is calculated from time step k − n through k. n is the number of time steps in the assimilation window and is chosen by the user. In the cost function, all observations between time steps k − n and n are taken into account. Contrary to the Kalman filter, variational assimilation does not directly provide an estimate of the error in the state estimate. We consider the following nonlinear system:

(16) $${\text{x}}_{\text{k}+1}={\text{g}}_{\text{k}+1,\text{k}}({\text{x}}_{\text{k}})$$

g_k+1,k_ is a nonlinear function relating the state at time step k to the state at time step k + 1. The system is observed as follows:

(17) $${\text{y}}_{\text{k}}={\text{h}}_{\text{k}}({\text{x}}_{\text{k}})+{\text{v}}_{\text{k}}$$

h_k_ is a nonlinear function relating the state at time step k to the observations at time step k. Note that variational DA considers not only measurements at time step k but also those at former time steps (until k − 1). The cost function J(x_k−n_) over the interval of n time steps is the following:

(18) $$\begin{array}{c} \text{J}({\text{x}}_{\text{k}-\text{n}})=\\ \frac{1}{2}{\left({\text{x}}_{\text{k}-\text{n}}-{\hat{\text{x}}}_{\text{k}-\text{n}}^{-}\right)}^{\text{T}}{\left({\text{P}}_{\text{k}-\text{n}}^{-}\right)}^{-1}({\text{x}}_{\text{k}-\text{n}}-{\hat{\text{x}}}_{\text{k}-\text{n}}^{-})+\frac{1}{2}\sum_{\text{i}=\text{k}-\text{n}}^{\text{k}}{\left[{\text{y}}_{\text{i}}-{\text{h}}_{\text{i}}({\text{x}}_{\text{i}})\right]}^{\text{T}}{\text{R}}_{\text{i}}^{-1}[{\text{y}}_{\text{i}}-{\text{h}}_{\text{i}}({\text{x}}_{\text{i}})] \end{array}$$

${\text{P}}_{\text{k}-\text{n}}^{-}$ is the background error covariance. A close inspection of the first term in the cost function shows that in effect one value indicating the error in the initial conditions is minimized. However, this formulation could be extended to include several terms, accounting for parameter calibration, or forcing terms, for example. If ${\text{P}}_{\text{k}-\text{n}}^{-}$ is equal to the identity matrix, this term is also equal to the Root Mean Square Error (RMSE) between the true state at time step k − n and the estimate of this state. If it is different to the unity matrix, the matrix ${\text{P}}_{\text{k}-\text{n}}^{-}$ can be considered as a weight factor. The second term in the cost function is again one value, indicating the mismatch between the observations and the simulations thereof. R_i_ is the uncertainty in the observations. If this is the identity matrix, this term is equal to the RMSE between the observations and the model simulations. If R_i_ is not equal to the identity matrix, it can again be considered a weight factor. The first term in the cost function is called the background error J_b_, and the second term is the observation error J_o_.

The objective of variational assimilation is the retrieval of the state x_k − n_ which minimizes this cost function. This can be achieved through optimization methods such as the Newton-Rapson method or the adjoint method. In the latter, the difference between y_i_ and h_i_(x_i_) for all time steps i between k and k − n is back-propagated in order to find the gradient in the cost function, which is then used to find the value for x_k−n_ for which the cost function is minimal.

Both model predictions and observations provide actual and important information on environmental state variables. Similar to EnKF and PF, VAR methods combine both information sources. They do not explicitly evaluate the large error covariance matrices which are propagated by Kalman Filters, but they simultaneously process the data within a given time period and implicitly take dynamic error information into account by propagating an adjoint variable [94]. By this procedure, VAR generates state estimates which consider uncertainties in the model, initial conditions and measurements. A Bayesian performance function is minimized by adjusting these uncertain elements with the goal of maximizing the accuracy. It can be implemented in an iterative procedure, which tends to converge for well-posed problems to vanishing mismatch between observed and simulated states [95]. In practice, it is stopped when some finite converge criterion is achieved. Another option involves stochastic methods [96,97]. VAR has already been applied in meteorological [98] and oceanographic [99] simulations for long periods. In terrestrial sciences, it has been used much less, but in groundwater hydrology, the inverse problem is often solved using a formulation which is in fact a VAR approach (e.g., Carrera and Neumann [100]). The main differences with the VAR approach in atmospheric sciences are that parameters are updated instead of initial conditions, and that the method is applied in batch (and not sequential) mode focusing on reproducing a historical time series. Terrestrial VAR implementations have also been introduced in other study areas of terrestrial sciences, e.g., in hydrology [101,102], geology [103], sediment transport estimation [104], crop modeling [105] and energy balance simulations [106]. Very prominent applications are soil moisture assimilation [101,107–111], flood prediction [112] and crop production [105].

## 3. Data Assimilation across States and Scales

An analysis of the literature on DA showed that four major approaches exist based on the number of states that are being assimilated and their corresponding scales: (1) univariate single-scale DA (UVSS), (2) univariate multiscale DA (UVMS), (3) multivariate single-scale DA (MVSS), and (4) multivariate multiscale DA (MVMS). In the subsequent section, we will briefly present and define these approaches giving specific examples for each of them. In addition, the special case of multisource DA will be defined.

### 3.1. Univariate Single-Scale Data Assimilation (UVSS)

Most publications about DA applications deal with the assimilation of a single data type (“univariate”), for which it is assumed that the scale at which it is measured coincides with the computational grid scale (“single-scale”). We define these approaches as univariate single-scale DA (UVSS). It is important to realize that although the measurement scale generally does not coincide with the computational grid scale, the scale mismatch is often not very large and is therefore neglected in DA study. Typically, support scales of observed environmental states are relatively small (e.g., a few cm^3^ to dm^3^) and are often several factors smaller than the model grid scale. If these observations are assimilated into a model with a grid size of tens of meters, the difference in the spatial scale is significant. In typical UVSS DA schemes, the observations are assumed to represent the average value of the observed values of the state within the model pixel without using an appropriate data scaling technique. For example, in a small catchment, a soil moisture sensor network has been installed with several sensors in vertical and horizontal directions. Usually, the soil volume measured by a sensor is just a few cm^3^. These observations are then assimilated into a 3D hydrological model with a spatial grid of 1 m^3^ in order to update the modeled soil moisture. Here, the spatial heterogeneity within one model grid element is neglected and it is assumed that the observation at the level of a few cm^−3^ is valid for the whole grid element of 1 m^3^. As the scale discrepancy was neglected and no scaling technique was applied, this example refers to UVSS DA. UVSS DA is not further discussed in this paper. However, reviews can be found, e.g., in Evensen [61], Moradkhani [4], Han and Li [45], McLaughlin [113] and Bocquet *et al*. [89].

### 3.2. Multivariate Single-Scale Data Assimilation (MVSS)

MVSS DA refers to the simultaneous assimilation of observation data for multiple model state variables into a simulation model. In these studies, the measurement data are either of the same scale as the computational grid, or, more commonly, scale disparities are neglected. The availability of simultaneous multiobservation pairs is an important characteristic. For example, leaf area index (LAI) and surface temperature can be obtained on the same spatial scale at the same moment by the MODIS satellite. Both data types can be assimilated in a multivariate and single-scale manner. The assimilation of remotely-sensed soil moisture and soil temperature is another example of MVSS DA. However, although the same assimilation moment is not mandatory for MVSS DA, it is important that the assimilation takes place in a certain time window. Assimilating soil moisture data from a microwave satellite, which overpasses an area under investigation at 06:00, and soil temperature data from a multispectral/thermal sensor, which overpasses at 10:00, into an hourly hydrological model would still require multivariate DA. This problem is usually solved by an augmented state vector, which is an important characteristic of MVSS DA. This updates only that part of the augmented state vector for which a corresponding observation is available. In contrast, calibrating a model by soil moisture DA in the first year and updating soil temperature by DA in the second year would not necessarily be characterized as multivariate DA, as we have defined it if the basic state vector is used.

If the DA framework can update both states and parameters, time series measurements of model parameters can also be assimilated. Therefore, our definition of MVSS DA is: (i) the assimilation of measurements for at least two model state variables, or (ii) at least one state variable in combination with at least one model parameter, or (iii) at least two different model parameters, at least one of which has the form of time series. For example, in several hydrologic models, LAI is a model parameter and soil moisture is a state variable. Both are time series products made available by satellite remote sensing. In a state-parameter estimation framework, where LAI is a parameter to be estimated, multivariate DA can be performed by updating both the soil moisture state and the LAI parameter.

### 3.3. Univariate Multiscale Data Assimilation (UVMS)

UVMS DA refers to the assimilation of external data obtained at a significantly different resolution than the model resolution and the application of a scaling technique. In multiscale DA, a technique is required to consider statistical parameters on all scales, such as observation and model error noise variances. Examples are the assimilation of coarse-scale soil moisture contents or snow water equivalents (which are disaggregated to the fine scale) into a fine spatial scale hydrologic model. The application of a scaling technique is mandatory to distinguish between the multiscale and single-scale DA applications described above.

Multiscale definitions given in other publications, which are not in line with the UVMS DA characteristics presented in this review, should however also be taken into account. We define UVMS DA as (i) the assimilation of a certain data type measured at a scale that is different to the computational grid scale, where DA explicitly takes into account this scale mismatch, or (ii) the assimilation of a certain data type measured at two or more different spatial scales, where DA explicitly takes into account that measurements were made at different scales. In the literature, other definitions can be found. For example, Lu *et al*. [114] define a process that assimilates both parameters and state variables as multiscale assimilation. However, this is not necessarily multiscale DA, as parameters and states could be measured at the same spatial scale. This definition therefore does not imply that DA solves the scale mismatch [48]. Another definition is given by Montaldo and Albertson [115]. They perform a multiscale DA by updating the root zone moisture to provide a temporal trajectory of the near surface moisture that follows the trajectory of the observed surface soil moisture, whereas the hydraulic conductivity is adjusted on the basis of the time-averaged corrections applied to the root zone water content. We argue that this is not multiscale DA, but an updating procedure that is nowadays inherent to modern DA techniques. Several definitions exist which flexibly consider the multiscale issue in the temporal or spatial domain. For example, the scale denotes the temporal resolution when measurements associated with different temporal resolutions are used, as published in Lu *et al*. [116] and Montaldo and Albertson [115]. This is an additional interesting aspect, but not the focus of this paper. Most measurements are point measurements in time and therefore we believe that the multiscale issue in the temporal domain is not as important an issue for most hydrological DA studies as the multiscale issue in the spatial domain [117].

### 3.4. Multivariate Multiscale Data Assimilation (MVMS)

MVMS DA refers to the complex combination of multivariate and multiscale DA techniques as defined above.

### 3.5. Multisource DA

In principle, in MVSS and MVMS DA, the state variables are updated by data sets from different sensors. Moreover, in most cases the multiscale issue in UVMS and MVMS is addressed by different sensors. However, a special case is imaginable, where one state variable is updated at a single scale by two different data sets obtained from different means of observation. In such a case, we recommend introducing an explicitly multisource UVSS DA. Multisource UVSS DA involves the assimilation of equal-scale soil moisture products from specific radar on two different satellites, or two different radar types on one single satellite. The advantage of such a multisource UVSS DA application is that different observation errors can be overcome, *i.e.*, one sensor performs better in one region, whereas the other sensor performs better in another region.

An example would be the assimilation of soil moisture products from the ERS 1 and ERS 2 satellites during their tandem phase. Two soil moisture products recorded at roughly the same time and at the same spatial resolution are provided. Such a simultaneous assimilation would not involve the DA procedures defined previously, *i.e.*, MVSS, UVMS, MVMS. In this case, it would involve multisource UVSS DA.

## 4. Univariate Multiscale Data Assimilation (UVMS)

The multiscale problem has been addressed by several different approaches because a wide range of natural processes have multiscale properties in space and/or time [118,119]. In this section, we will focus on the assimilation of one state variable (or in certain cases, one parameter) obtained by (i) observation systems operating at two or more spatial scales into terrestrial models or (ii) the combination of one or more observation systems and a modeling grid at different spatial resolutions.

### 4.1. Methodology

Both the PF and the EnKF are excellent algorithms that assimilate data obtained at a certain spatial resolution into models that operate at a different resolution. This can be performed in two ways. The first approach is to use the observation operator [Equation (2)]. The second approach is to rescale the observations to the model scale prior to assimilation. In the following, both methods are briefly explained.

#### 4.1.1. Use of the Observation Operator

**Figure 3.**
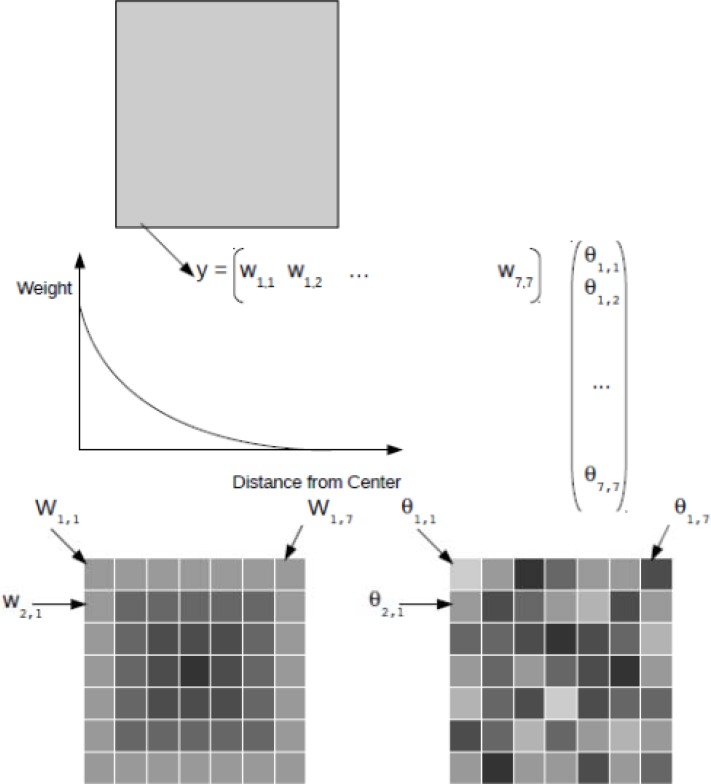
Schematic of the use of the observation operator for the assimilation of coarse-scale data into a fine-resolution 2D model. w_i,j_ stands for the weight of the model result in row i and column j in the calculation of the grid-averaged model result. Darker colors represent higher weights. θ stands for the model results, and darker values represent higher values. For simplicity, the .^−^ is omitted from the y and θ variables, and the time index k is omitted from all variables.

Figure 3 shows a schematic of how the observation operator can be used to assimilate coarse-scale data into a 2D fine-resolution model. In this methodology, the observation operator h_k_(.) [Equation (2)] uses the modeled state variables to simulate the large-scale observation. A typical example is the assimilation of coarse-scale Soil Moisture and Ocean Salinity (SMOS) data into a fine-resolution hydrologic model. It is straightforward to prove that the antenna configuration causes locations close to the center of an SMOS grid to contribute more to the observed signal than locations further away from the center [120]. In the simulation of the SMOS signal (using the fine-scale hydrologic model results), the pixels in the center of the SMOS grid should thus receive a higher weight. The simulated large-scale signal (one single value) is then used to update the soil moisture contents of all hydrologic model pixels inside the SMOS grid.

If the EnKF is used, the impact of the different weights can be assessed by examining the update equations. Let us assume that the hydrologic model is column-based, which means that the model results of all modeled pixels are independent of each other. This is a common feature of many hydrologic models, such as the widely used Community Land Model [121]. Let us also assume that the uncertainties are identical in the model results for the different pixels.

Under these conditions ${\text{P}}_{\text{k}}^{-}$ is the identity matrix multiplied by the variance in the modeled results calculated over the ensemble. In this case h_k_(.) is a linear function (a linearly weighted average is obtained in order to simulate the large-scale observation), and can be written as the H_k_ vector (the vector containing all the weights). The denominator in the Kalman gain equation is a single variable. Since ${\text{P}}_{\text{k}}^{-}$ is a uniform diagonal matrix, the magnitude of the Kalman gain for each state variable will be determined by the value of its entry in H_k_, the weight matrix. In other words, pixels with a higher weight will receive a larger update than pixels with a lower weight. A limiting case may occur when the weight of a certain pixel is zero, which implies that its value does not contribute to the large-scale signal. In this case, the requirement of observability of the system is violated and the pixel should be left out of the analysis.

The impact of the averaging weight is fundamentally different when a PF is applied. The large-scale observation is simulated in exactly the same way as for the EnKF. However, in this case, one single particle contains the states (and possibly parameters) at all the modeled pixels, and is one of the ensemble members of the model realizations. Each particle possesses its own weight. The adaption of the weight depends on the deviation of the simulation of the large-scale observation from the actual observation. The particles could then be resampled. However, in this case, the modeled state variables for a certain particle are simply duplicated. In other words, there is no differential update in contrast to the EnKF.

VAR deals with this problem in another way. An initial state vector is eventually retrieved that minimizes the cost function. Since the pixels located near the center of the large-scale grid have the highest weight, their state estimate will match the truth better than the pixels further away from the center. The difference compared to the EnKF is that these results are not obtained through an update, but through a minimization of a cost function. The same reasoning can be applied when an entire profile needs to be updated instead of one single layer. The difference is that the matrix H_k_ will contain zeros for all state variables that are not in the uppermost layer of the profile.

#### 4.1.2. Prior Downscaling

**Figure 4.**
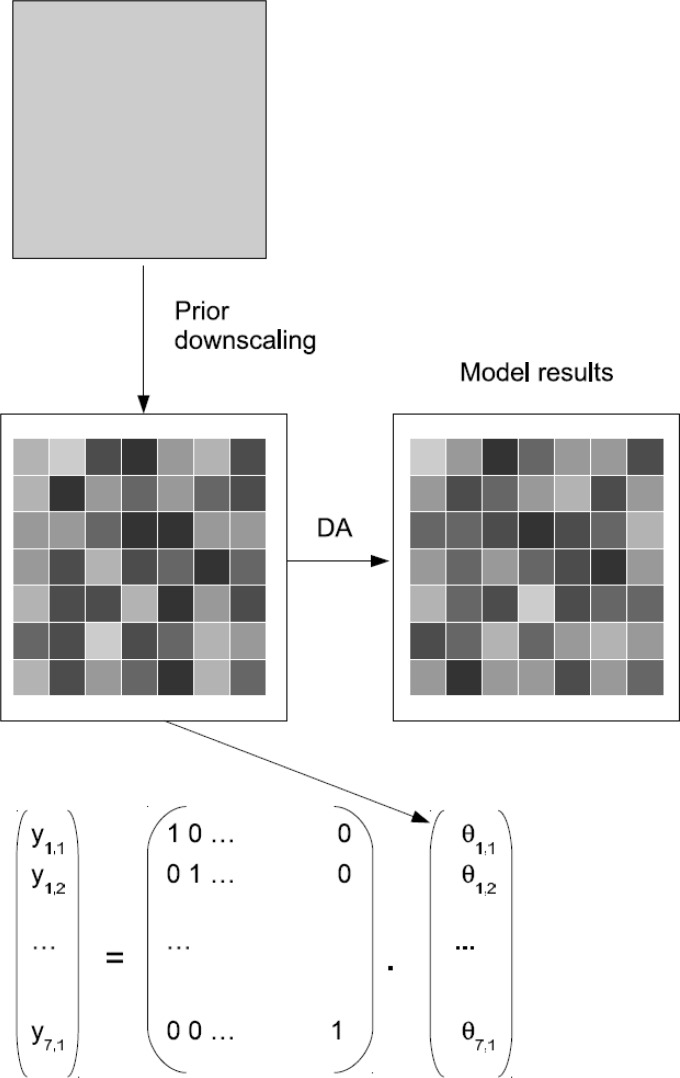
Schematic of the use of prior downscaling for the assimilation of coarse-scale data into a fine-resolution model for a model with only one model layer. The symbols are identical to those in Figure 3. As in Figure 3, the .^−^ is omitted from the y and θ variables, and the time index k is omitted from all variables. DA refers to data assimilation.

A different approach involves downscaling the observations to the spatial resolution of the model before the observations are assimilated. Figure 4 shows a schematic of this approach for a model with one model layer. In this case, the dimension of the observation vector y_k_ is the same as the number of pixels inside the large-scale grid. H_k_ is the identity matrix. The downscaled model results can then directly be assimilated into the fine-scale model. In some applications, only one layer might be observed (e.g., from remote sensing), but multilayer systems need to be updated. In such cases, H_k_ again contains zero values for all model variables that are not located in the uppermost layer.

A drawback of this methodology is the need for a downscaling algorithm and the quantification of the measurement uncertainty on the fine scale. On the other hand, the advantage is the straightforward application of the DA algorithms, especially when multiple data sets at different spatial resolutions need to be assimilated.

This is also true for the assimilation of one state variable obtained on two or more spatial scales into terrestrial models. An example would be the assimilation of fused top-soil-moisture products obtained by active (relatively higher spatial resolution) and passive (relatively lower spatial resolution) microwave methods. In addition to the prior individual downscaling of both data sets to the model resolution, prior data fusion could also be feasible. A huge range of methods have been published in relation to satellite image fusion [122,123]. However, if the physical information of the data set is to be conserved, well-established techniques, such as Intensity-Hue-Saturation (IHS) transformation [124], Brovey Fusion [125], and Principal Component Fusion (PCA) [126], cannot be used for DA. Here, the coarse-scale data set is transferred into a new data system (e.g., IHS or PCA) with several components, one component is substituted by the fine resolution data set, and the transfer procedure is inverted. This results in a combined fine-resolution data set, but the absolute values are heavily altered. In contrast, the wavelet theory provides several options for an application in multiscale terrestrial DA [127–129]. In principle, the method published by Das *et al*. [130] for the multiscale fusion of active and passive microwave data obtained by the SMAP mission [131] is a wavelet-based approach.

### 4.2. Applications

#### 4.2.1. Univariate Dual-Scale Data Assimilation

Several studies have been published assimilating one state variable at a specific spatial resolution to a model on another spatial resolution. As a simple method of transfering the spatial differences in soil moisture observations from ASCAT (∼25 km) and AMSR-E (∼38 km) to the model grid of 25 km, Draper *et al*. [132] used a simple nearest neighbor interpolation. Here, the scale difference is not large which could justify the approximation made. McLaughlin [133] addressed the interpolation problem in hydrological DA using a multiplicative cascade model for statistical downscaling prior to assimilation. Reichle *et al*. [101] used 4D-VAR to constrain a physically based soil-vegetation-atmosphere (SVAT) model with surface soil moisture and L-band brightness temperature measurements. One goal was to derive soil moisture estimates on a finer scale based on coarse-scale satellite observations. The downscaling procedure made use of a simple arithmetic average to reflect the transfer of information from observation pixels (at larger scales) to nested estimation pixels (at smaller scales). Li *et al*. [134] coupled the EnKF with a statistical upscaling in order to introduce fine-scale hydraulic conductivity data into a coarse-scale groundwater flow and mass transport model. Merlin *et al*. [135] assimilated simulated SMOS observations to a distributed SVAT model with the EnKF. In principle, two data sets are used for the assimilation of one state variable. Prior downscaling was performed with fine-scale soil skin temperature observations from thermal/optical sensors describing the spatial variability of surface soil moisture within the simulated SMOS pixel. The disaggregated soil moisture is calculated by inserting the fine-scale skin temperature residuals into the coarse-scale SMOS soil moisture product. Using this observation operator, the observation and the model have similar spatial scales.

Parada and Liang [136] used the multiscale Kalman filtering (MKF) technique based on the data fusion method by Chou *et al*. [137] for the assimilation of near-surface soil moisture fields derived from an electronically scanned thinned array radiometer (ESTAR) into the three-layer variable infiltration capacity (VIC-3L) land surface model [138]. VIC-3L was calculated at a resolution of 1/32° (approx. 3.2 km), whereas the ESTAR retrievals were recorded at a resolution of 800 m. To fully describe the spatial dependence of near-surface soil moisture with the MKF-based DA approach, both the land surface model predictions for near-surface soil moisture and the remotely sensed imagery are treated as observation sources with individual observation noise terms. This accounts for the fact that the degree of uncertainty in the predictions from both land surface models and observations vary over time. The MKF captures the persistent spatial dependence of soil moisture over large distances and it further and better constrains the optimal state estimates. Future studies may exploit this concept by integrating additional multiscale data sources of the same state variable into the system. Moreover, the ability to account for the presence of bias in the model as well as the observations may support the use of this method for the multiscale assimilation of soil moisture. An Expectation Maximization Algorithm [139] can be used to obtain time-varying statistical parameters to describe how the uncertainty in the observed and the modeled soil moisture may change over time. As radiometer-based soil moisture estimates refer to the top few centimeters of soil only [14,140], model predictions and no observations exist for the deeper soil layers. Without additional observations, however, we can say that the soil layers 2 and 3 of VIC-3L are single-scale hidden states. In order to additionally update these single-scale states, a conditioning function can be specified based on the underlying physics describing soil moisture dynamics in the VIC-3L land surface model. An important aspect of this approach is the ability to retain mass conservation at all scales, so that the mean near-surface and deeper layer soil moisture content is preserved from scale to scale.

Frakt and Willsky [141] introduced the multiscale autoregressive (MAR) framework. The advantage of MAR is the replacement of a high-dimensional filtering problem with low-dimensional localized filtering problems defined across scales. The method was later established by Zhou *et al*. [142] in an ensemble form, *i.e.*, the ensemble multiscale filter (EnMSF). Pan *et al*. [118] introduced the EnMSF to hydrological land surface-driven applications, accounting for the horizontal coupling in surface hydrology, and the subsequent horizontal error correlations in measurements and state variables. Horizontal coupling allows the filter to update one pixel based on measurements of other pixels. More research on UVMS DA is needed to adequately address the horizontal coupling issue and to extend it to spatial error correlations. Pan and Wood [143] further analyzed the impacts of accuracy, spatial availability and assimilation frequency (*i.e.*, satellite re-visit time) on this assimilation framework. A multiscale tree topology of states is generated by dividing a root node into different clusters of child nodes, which are again divided into different clusters. Once a coarse observation is available, it is recursively clustered to the finer computing grid using the tree topology. With a synthetic experiment, Burke *et al*. [144] presented an approach to assimilate coarse-scale brightness temperature to a coupled soil-water-evaporation and transpiration (SWEAT) and microwave emission (MICRO) model on a finer scale. They calculated the brightness temperature using Mesonet data and generated coarse-scale simulated observations by area averaging. They then reduced the precipitation by 40% and re-calculated the brightness temperature on a finer resolution. The latter was updated by the simulated observations by comparing the area average. This technique does not alter the relative differences between the model grid squares of the finer scale, but the result is de-biased. Here, brightness temperature observations from satellites without any bias are mandatory. So far, this is not the case for SMOS for specific regions [145–147].

Another synthetic experiment was performed by Hill *et al*. [148]. They used the Monte Carlo Metropolis-Hastings Sequential Particle Filter as proposed by Dowd [149] to assimilate Normalized Difference Vegetation Index (NDVI) using a model operator translated to LAI. The net ecosystem exchange of carbon was calculated from respiration and gross primary production estimates. The coarse resolution observations were combined with an estimate of their subpixel probability distribution function and fine-resolution model states. The resulting fine-resolution disaggregated observations were then assimilated with standard procedures.

#### 4.2.2. Univariate Multiscale Data Assimilation with Prior Fusion or Downscaling

In order to meet the problem of assimilating two or more observation data sets at dissimilar spatial scales, these observations are often fused prior to the assimilation. For example, in a synthetic study in the Arkansas-Red River Basin, Dunne *et al*. [80] generated “true” soil moisture states at a resolution of 1 km with the topographically based land-atmosphere transfer scheme (TOPLATS) model [150]. Simulated active and passive microwave observations were generated using the Microwave Emission and Backscatter Model. These synthetic observations were aggregated to meet the mission concept of the Soil Moisture Active and Passive (SMAP) Mission [131], *i.e.*, 36 km for brightness temperatures and 3 km for backscatter data. The Noah Land Surface Model was used for assimilation at a spatial resolution of 6 km. Hence, three spatial scales are present in the DA system (3 km, 6 km, and 36 km). A transformation matrix is created to relate the predicted measurements on the Noah model scale to the observations of brightness temperature and backscatter at their respective resolutions. The brightness temperatures predicted at 6 km were linearly averaged within each 36 km radiometer grid cell for comparison with the observed brightness temperatures. Each 6 km predicted backscatter measurement was transformed into four collocated 3 km predicted backscatter values for comparison with the radar observations [80]. This information was then assimilated into the Noah model by an Ensemble Kalman Smoother (EnKS).

Wang *et al*. [117] further developed the multiscale Kalman Smoother-based framework by Parada and Liang [136] in order to fuse precipitation data from different data sets. The intention was to consider the individual noise characteristics and biases of each data set. The study focused on combining data sets with different spatial resolutions. It combined precipitation as recorded by *in situ* rain gauges, ground-based US Next-Generation Radar (NEXRAD) network and the PERSIANN system, which already combines the Tropical Rainfall Measuring Mission (TRMM), the Microwave Imager (TMI) and the Geosynchronous Satellite Longwave Infrared Imagery (GOES-IR). The multiscale Kalman Smoother (MKS) algorithm can be used flexibly in a time or space domain. In order to achieve the same estimated mean of the fused precipitation at all scales, a bias compensator was introduced. This bias compensator minimizes the impacts of inconsistencies (e.g., biases) between measurements at different scales on the fused precipitation. The method makes use of an upward sweep followed by a downward sweep, where information in multiscale measurements collectively propagate to all the nodes of the multiscale tree, as expressed by the root on the coarse scale and the leaves on the fine scale. Via the upward sweep, the finer-resolution data add their influences to the estimates of the hidden states at coarser resolutions. Via the downward sweep, the coarser-resolution data add their influences to the estimates of the hidden states at finer resolutions. During this downward sweep, the estimates of the hidden states are further refined using a scale-recursive Kalman smoothing step.

## 5. Multivariate Single-Scale Data Assimilation (MVSS)

### 5.1. Methodology

The EnKF, and variants of it, were used more often than PF and variational methods for multivariate DA in hydrology. For EnKF, the different observation types are all grouped together in the vector y_k_. Therefore, compared to the univariate case, the vector y_k_ is “extended” to include different types of measurements. The state vector x_k_ also includes different types of state variables, and possibly parameters as well. These different variables and parameters are included in the state vector in the form of blocks: a first block for the first state variable, then additional blocks for additional state variables, and finally blocks for the different parameters. The covariance matrix is therefore also extended and includes cross-covariances between different state variables, or cross-covariances between a state variable and a parameter. The update equation for EnKF, which is valid both for univariate and multivariate DA, is:

(19) $${\text{x}}_{\text{k}}^{\text{i}+}={\text{x}}_{\text{k}}^{\text{i}-}+[{\text{P}}_{\text{k}}^{-}{\text{H}}_{\text{k}}^{\text{T}}{\left({\text{H}}_{\text{k}}{\text{P}}_{\text{k}}^{-}{\text{H}}_{\text{k}}^{\text{T}}+{\text{R}}_{\text{k}}\right)}^{-1}]\;({\text{y}}_{\text{k}}-{\text{H}}_{\text{k}}{\text{x}}_{\text{k}}^{\text{i}-})$$

We will now look at the example of two state variables, which were modeled and observed. Moreover, two distributed parameter fields were calibrated, and observations were available for one of the parameters. The augmented state vector, observation vector and covariance matrix are now composed of the following blocks:

(20) $${\text{x}}_{\text{k}}^{\text{i}}=\left\{\begin{array}{l} {\text{x}}_{s_{1}}\\ {\text{x}}_{s_{2}}\\ {\text{x}}_{p_{1}}\\ {\text{x}}_{p_{2}} \end{array}\right\};\;\;\;\;\;\;\;\;\;\;{\text{y}}_{\text{k}}=\left\{\begin{array}{l} {\text{y}}_{s_{1}}\\ {\text{y}}_{s_{2}}\\ {\text{y}}_{p_{1}} \end{array}\right\};\;\;\;\;\;\;\;\;\;{\text{P}}_{\text{k}}^{-}=\left[\begin{array}{llll} {\text{P}}_{s_{1}s_{1}} & {\text{P}}_{s_{2}s_{1}} & {\text{P}}_{p_{1}s_{1}} & {\text{P}}_{p_{2}s_{1}}\\ {\text{P}}_{s_{1}s_{2}} & {\text{P}}_{s_{2}s_{2}} & {\text{P}}_{p_{1}s_{2}} & {\text{P}}_{p_{2}s_{2}}\\ {\text{P}}_{s_{1}p_{1}} & {\text{P}}_{s_{2}p_{1}} & {\text{P}}_{p_{1}p_{1}} & {\text{P}}_{p_{2}p_{1}}\\ {\text{P}}_{s_{1}p_{2}} & {\text{P}}_{s_{2}p_{2}} & {\text{P}}_{p_{1}p_{2}} & {\text{P}}_{p_{2}p_{2}} \end{array}\right]$$

where *s*_1_ corresponds to the first state variable, *s*_2_ to the second state variable, *p*_1_ to the first parameter and *p*_2_ to the second parameter. It should be noted that the dimensions of the blocks normally differ between the augmented state vector and the observation vector, as not each modeled state is observed. The Kalman gain [see also Equation (5)] then determines the relative weights for each of the different measurements to update each of the state variables and the two parameter values at each grid cell. DA methods related to EnKF, such as the extended Kalman filter (EKF), the ensemble Kalman smoother or the ensemble smoother, can perform multivariate DA in a similar way. In practice, the complete covariance matrix is not calculated; instead, only the cross-covariances between observed grid cells and other model states and parameters are calculated.

Variational methods have also been used in several cases for multivariate DA in hydrology. Multivariate DA with variational methods is performed by evaluating the second term on the right-hand side of Equation (18) for all observations. However, a key role is played by matrix R_i_ in this expression. This matrix contains the estimated measurement error variances for each of the observations. The different types of observations are associated with different uncertainties and the diagonal elements of *R_i_* will therefore have different values. The matrix R_i_ weights the influence of the different observations to update the simulated model values with the observations. The correcting influence of the observations also depends on the values in R_k_ compared to the covariance matrix for the background errors, P_k_. The inclusion of additional observations, and the comparison of the measured values with the simulated ones, according to Equation (18), makes the calculation of the gradient of the objective function more cumbersome. For example, if observations for additional state variables are included, the gradient of the objective function with respect to those state variables should also be calculated.

The PF has not yet been used frequently for multivariate DA in hydrology. Different data sources can be relatively easily included as conditioning information in the particle filter, which can be understood by inspecting the likelihood function. The likelihood for the multivariate case is obtained by comparing the different measurement data with their simulated equivalents, and weighting each of the residuals with the measurement variance. For the univariate case, the probability of the observations in the modeled state was given by Moradkhani *et al*. [48] as:

(21) $$\text{p}({\text{y}}_{\text{k}}|{\text{x}}_{\text{k}}^{\text{i}})=\frac{L({\text{y}}_{\text{k}}|{\text{x}}_{\text{k}}^{\text{i}})}{\sum_{\text{i}=1}^{N_{p}}L({\text{y}}_{\text{k}}|{\text{x}}_{\text{k}}^{\text{i}})}$$

where *N_p_* is the number of particles and *L*(*y*|*x*) is the likelihood of the observations given the modeled states for a particle:

(22) $$L({\text{y}}_{\text{k}}|{\text{x}}_{\text{k}}^{\text{i}})=\frac{1}{{\left(2\pi \right)}^{\frac{1}{2}}{\left({\text{R}}_{\text{k}}\right)}^{\frac{1}{2}}}\times \text{exp}\left(1-\frac{1}{2{\text{R}}_{\text{k}+1}}{\left({\text{y}}_{\text{k}}-{\text{H}}_{\text{k}}{\text{x}}_{\text{k}}\right)}^{2}\right)$$

For the definition of R_k_ and H_k_, see Section 2.1 on EnKF. In these expressions, it was assumed that all observations have the same measurement error variance. For the multivariate case, the expression for the likelihood of the observation modifies to:

(23) $$L({\text{y}}_{\text{k}}|{\text{x}}_{\text{k}}^{\text{i}})=\frac{1}{{\left(2\pi \right)}^{\frac{1}{2}}|{\text{R}}_{\text{k}}{|}^{\frac{1}{2}}}\times \text{exp}\left(-\frac{1}{2}{\left({\text{y}}_{\text{k}}-{\text{H}}_{\text{k}}{\text{x}}_{\text{k}}\right)}^{\text{T}}{\text{R}}_{\text{k}}^{-1}({\text{y}}_{\text{k}}-{\text{H}}_{\text{k}}{\text{x}}_{\text{k}})\right)$$

We see in this expression that the measurement error variances are in matrix notation, which acknowledges that different measurement types will be associated with different uncertainties. Measurement errors for different observations can also be correlated in space, as could be the case for remote sensing data. The uncertainty of the different (types of) observations affects the weighting of the particles.

### 5.2. Applications

Although multivariate DA seems like a relatively straightforward extension of univariate DA, most studies in terrestrial systems assimilate only one data type. The complication of MVSS DA is not so much of an algorithmic nature, but is related to the specification of the measurement uncertainty for all data types involved. If different data types are assimilated, the correct weighting of the different pieces of information becomes very important for the efficiency of the procedure. The following discussion of the papers that deal with MVSS DA is organized according to the application area, focusing on developments during the last decade and on EnKF, PF and VAR.

#### 5.2.1. Groundwater

In groundwater hydrology, sequential DA focused from the outset on jointly updating states and parameters by assimilating piezometric head data using an augmented state vector approach. The work of Chen and Zhang was among the first in this area [74]. Initial work on multivariate DA considered the joint assimilation of time series of piezometric heads and conditioning to hydraulic conductivity data. In such publications, information on hydraulic conductivity was assimilated in the first step and later preserved in the DA [151–153]. The joint assimilation of measurements for more than one state variable is less frequently reported in the literature. Liu *et al*. [154] and Nowak [151] assimilated both piezometric head and concentration data, but they provided few details about the assimilation of the concentration data and the (expected) non-Gaussian concentration distribution. Li *et al*. [155] provided a more detailed analysis of the value of additional concentration data, and found that the joint assimilation of head and concentration data gave much better results than the assimilation of head data only. They used the classic EnKF and a very large ensemble size (1,000 realizations). In addition to head and concentration data, Li *et al*. [155] also assimilated hydraulic conductivity and porosity data, which are both model parameters. Schöniger *et al*. [156] pointed out that when concentration data is assimilated, EnKF is expected to perform suboptimally because local pdfs of concentration tend to be non-Gaussian, and simple univariate normal score transformations cannot be expected to render bivariate pdfs Gaussian.

#### 5.2.2. Integrated Surface-Subsurface Hydrology

There has been a recent increase in papers concerned with DA for partial differential equation-based coupled surface-subsurface models. Crow and van Loon [47] suggested that the joint assimilation of soil moisture and stream discharge data is one strategy of detecting erroneous assumptions about the magnitude of model errors. Camporese *et al*. [157] assimilated pressured head and streamflow data in CATHY [158–161] using EnKF. They concluded from the synthetic experiment that pressure head data are helpful for improving both the characterization of subsurface states and river discharge, whereas river discharge data do not improve the characterization of subsurface states. Pasetto *et al*. [51] further studied the joint assimilation of discharge and pressure head data in the CATHY model, this time using PF, but (again) without updating model parameters. They proposed a modification of SIR-PF for more efficient assimilation, and otherwise their conclusions were similar to those from Camporese *et al*. [157]; discharge data contributed little to improving the characterization of subsurface states. Bailey and Baù [162] had a slightly different focus and updated not only model states, but also hydraulic conductivities. They assimilated piezometric head measurements, groundwater return flow volume data and hydraulic conductivity data to estimate the spatially variable hydraulic conductivity field and they showed that the best results are obtained when all data are jointly assimilated. In their synthetic experiment, they used an Ensemble Smoother for assimilation, which considers current and past time steps together in the conditioning approach. In a later publication, Bailey and Baù [163] used the ensemble smoother in combination with CATHY to assimilate pressure head and water level data. They found that water level data are much more helpful for conditioning than streamflow discharge data. In their study, information on surface flow also contributed to an improved characterization of subsurface hydraulic conductivities. An additional feature of their synthetic study was that geostatistical parameters of the subsurface were also made uncertain.

#### 5.2.3. Rainfall Runoff

Some authors used more conceptual hydrological models for assimilation focusing on the reproduction of river discharge. In an early publication, Seo *et al*. [164] used the Sacramento model [165,166] and VAR to assimilate discharge data, precipitation and potential ET. However, precipitation and potential ET were not assimilated in the classical sense, but were incorporated as observed model forcings. Lee *et al*. [167] used SACRAMENTO to assimilate discharge and soil moisture data with VAR. Their conclusions were very similar to the ones drawn by Camporese *et al*. [157] and Pasetto *et al*. [51], but they found that river discharge data also improved the characterization of subsurface states in the synthetic case to some degree. Interestingly, this was not the case for the real-world case study of the Eldon catchment (Oklahoma and Arkansas, USA). The authors assumed perfectly known forcings and no measurement errors in both the synthetic and real-world cases, which is unrealistic for the real-world case. Xie and Zhang [168] used SWAT [169–171] to assimilate runoff, soil moisture and evapotranspiration data with EnKF and they updated various model parameters. This was also a synthetic study, and it was assumed that soil moisture and evapotranspiration was known for complete sub-catchments. In this very optimistic setting, best results were again obtained when all data were jointly used for assimilation, but evapotranspiration data contributed less than the other types of measurements.

#### 5.2.4. Vadose Zone

The applications of DA in vadose zone hydrology are traditionally concerned with the assimilation of remote sensing data. In these applications, normally only states are updated, while for soil hydraulic parameter calibration, inverse methods are used. DA for vadose zone hydrology has a strong link with land surface hydrology and we distinguish here between assimilation experiments for single soil columns (vadose zone hydrology) and studies for larger areas with distributed land surface models (land surface hydrology). Although Walker *et al*. [172] already carried out synthetic experiments for the assimilation of soil moisture and surface temperature data from satellite into vadose zone models, the joint assimilation of these data was not considered in this publication or in the years that followed. Visser *et al*. [173] assimilated both measured groundwater level and soil moisture data (De Bilt, The Netherlands) in a model for flow in the unsaturated zone and drainage. They used a simplified form of Newtonian nudging in their experiments. In Newtonian nudging, deviations between model prediction and measurements are included as additional sink and source terms in the equation. They showed that DA can reduce the errors for soil moisture prediction, and that best results were obtained when soil hydraulic parameters were also calibrated. They found that the model error had the greatest influence on the prediction error.

#### 5.2.5. Large-Scale Land Surface

One option for improving the prediction quality of larger-scale land surface models is the joint assimilation of soil moisture and surface temperature data. Barrett and Renzullo [174] developed nonlinear measurement operators for the assimilation of satellite-measured brightness temperature and land surface temperature. They then investigated the conditions under which the joint assimilation of these data could be useful. Brightness temperature always puts a strong constraint on soil moisture content, but surface temperature data can only put additional constraints on soil moisture content estimates if: (i) the measurement error is not too big (around 1 K), (ii) the background error on soil moisture contents is relatively large (e.g., 5 vol%), and (iii) the sensitivity of surface temperature with respect to soil moisture is increased (which is the case for larger LAI). Han *et al*. [175] tested the joint assimilation of brightness temperature and surface temperature in a synthetic study and their conclusions agree well with those of Barrett and Renzullo [174]. The joint assimilation only improved the results marginally compared to the assimilation of one data type only. Another way of improving the prediction quality of larger-scale land surface models is the joint assimilation of soil moisture data and LAI. Pauwels *et al*. [176] used the land surface model TOPLATS [177] coupled with the crop growth module of WOFOST [178] to assimilate these data with EnKF. They found in a synthetic study that soil moisture data help to improve the estimates of LAI during the growth season, whereas LAI hardly improved soil moisture characterization. The best results were again obtained when both observation types were jointly assimilated, and for LAI it was important that data were assimilated in a biweekly interval. Sabater *et al*. [179] reached similar conclusions when applying a land surface model simulating crop growth to data from a field site close to Toulouse in France. They used a 1D VAR approach to jointly assimilate LAI and soil moisture data. Albergel *et al*. [180] studied the joint assimilation of soil moisture and LAI at the same site in the ISBA land surface model [181–184] using the EKF. The joint assimilation resulted in improved estimates of water, energy and carbon fluxes. Draper *et al*. [185] analyzed the assimilation of remotely sensed soil moisture from AMSR-E as well as temperature and relative humidity measured at 2 m in the land surface model ISBA using the EKF. Both data products resulted in very different updates of soil moisture and the joint assimilation was not very different from the assimilation of temperature and relative humidity alone. It was concluded that AMSR-E could not constrain the soil moisture estimates very much.

DA might violate the mass balance because at each assimilation time step, mass might be removed or injected into the system. Pan and Wood [186] proposed the Constrained Ensemble Kalman Filter (CEnKF) to put additional constraints on the mass balance and to avoid large mass imbalances. This method is especially interesting in combination with different kinds of observations that allow the mass balance to be constrained. Pan and Wood [186] assimilated soil moisture data, latent heat estimates and stream flow into the VIC land surface model [138,187,188]. The authors also demonstrated that soil moisture data can be used to constrain precipitation measurements with this methodology. Although this involved a multivariate DA, Pan and Wood [186] did not focus on the value of the different pieces of information in this paper.

#### 5.2.6. Snow Hydrology

In the area of snow hydrology, multivariate DA has also been explored as a method of improving the characterization of snow pack and runoff estimates during snow melt. Durand and Margulis [189] investigated the potential of remotely sensed snow observations and combined the Special Sensor Microwave Imager (SSM/I), 12 channels of the Advanced Microwave Scanning Radiometer-Earth Observing System (AMSR-E) and broadband albedo observations to improve the estimation of snow water equivalent (SWE) using EnKF as a DA technique. They found a strong improvement in the characterization of SWE, with a RMSE as low as 3 cm (for a total maximum SWE of 80 cm). Pullianen [15] also assimilated SWE from AMSR-E and SSM/I in combination with *in situ* snow depth measurements and used a Bayesian approach. The approach was tested for areas in Finland, Russia and Canada. It was found that a combination of different data sources gave the best results. Pullianen [15] introduced a forward modeling approach for the brightness temperature. Kolberg *et al*. [190] assimilated a remotely sensed snow-covered area (from Landsat 7 ETM+ images) and related this via a simple model to SWE. The combined assimilation of remotely sensed snow-covered area and river discharge gave the best results. The time of assimilating the Landsat 7 ETM+ images had an important impact on the results, and the best results were generated when the assimilation was performed shortly before the main flood peak.

## 6. Multivariate Multiscale Data Assimilation (MVMS)

Studies presenting a complex combination of multiscale (Section 4) and multivariate (Section 5) DA techniques are discussed in the following. Most of the MVMS DA studies deal with the assimilation of snow data and the assimilation of soil moisture and surface temperature. Durand *et al*. [191] assimilated synthetic 25 km snow water equivalent (SWE) and 1 km snow grain size data to a land surface model with a resolution of 1 km using adaptive EnKF. They examined the impacts of different uncertainties on the efficiency of the snowpack characterization in DA. The results showed that the SWE and grain size estimation efficiency were both influenced by the snow grain size and precipitation accuracy and were related to the coefficient of variation and correlation length for both the precipitation and grain size. De Lannoy *et al*. [192] used the snow water equivalent (SWE) from 25 km resolution AMSR-E products and a snow cover fraction (SCF) from 500 m resolution MODIS products to improve the snow water equivalent estimation in the Noah model (resolution 0.01 degree) with a multiscale EnKF framework. The coarse SWE data were assimilated with the aggregated model simulation, and the high-resolution SCF data were aggregated to the model resolution and were transformed to SWE using the snow depletion curve as the observation operator. A rule-based update of SWE was used for SCF. The results showed that the joint assimilation of SWE and SCF improved the model estimation significantly for areas with shallow snow packs. For the deep snow areas, the results were not improved significantly because of the bias in the coarse SWE products and the assimilation of SCF only had a marginal impact. Su *et al*. [193] studied the impact of assimilating the GRACE terrestrial water storage (TWS) and MODIS snow cover fraction (SCF) data into the Community Land Model. The ensemble Kalman filter and smoother were used and the joint assimilation yielded better estimates of SWE and snow depth than the MODIS-only approach.

Balsamo *et al*. [194] used an observation system simulation experiment (OSSE) to study the impacts of microwave L-band and C-band, infrared surface temperature, screen-level temperature and relative humidity MVMS DA on the daily soil moisture and temperature analysis in a land DA system with a simplified VAR. In general, OSSE is designed to enable the modeler to examine the performance of data assimilation procedures and even to obtain the sensitivity of the procedure to different models with different parameterizations, observation operators and physical representations. The spatial coverage, temporal availability, and nominal or expected errors were considered using the present satellite observation information. The results showed that the observable with the largest dynamical response to perturbations of the model states contributed significantly to the analysis. Barbu *et al*. [195] studied the joint assimilation of the soil wetness index (SWI) and LAI through a simplified extend Kalman filter in the ISBA-A-gs land surface model. The results showed that the assimilation of surface-measured SWI could improve the root zone soil moisture significantly, and that LAI could correct a number of deficiencies in the model. The root mean square errors of CO_2_ fluxes were also reduced by about 5% with this joint scheme.

## 7. Advantages and Disadvantages of Multivariate and Multiscale Data Assimilation

Compared to UVSS DA techniques, multivariate and multiscale assimilation (MVSS, UVMS, MVMS) allow additional data containing information about the states to be updated and quantities of interest (e.g., fluxes) to be modeled. Advantages have been outlined in several studies [30,143]. However, multivariate and multiscale assimilation raises questions about the information content of these data.

### 7.1. Univariate Multiscale Data Assimilation (UVMS)

UVMS DA allows observations with different “support” scales to be integrated into mathematical models. However, this necessitates the availability of upscaling and downscaling approaches. One problem associated with UVMS DA is the assimilation of data measured at a certain scale into a model with a different grid resolution. It has been shown that assimilation is straightforward. For methods like EnKF, it can be handled by the observation operator, while PF can assimilate directly. Simulated values can be compared with measured values in a relatively straightforward manner: if for example the measurement comprises multiple computation grid cells, the observation operator can handle an equal weighting of all the measurement grid cells, while also taking account of an unequal weighting of the grid cells (as was the case in the SMOS example in Section 4.1). However, the problem is more complicated if a property scales nonlinearly. This is the case for example for brightness temperature measured by microwave sensors, which is nonlinearly related to soil moisture. When the relation between brightness temperature and soil moisture is applied to the larger-scale grid, we expect the soil moisture value to be different to the value we would receive if the brightness temperature was available for all smaller computational grid cells and if the conversion from brightness temperature to soil moisture was calculated for each of these grid cells. For properties that scale linearly, we expect the direct application of the observation operator (in the case of EnKF) to give the best results. For properties that scale nonlinearly, alternative strategies such as prior downscaling are promising. A systematic comparison of methods solving assimilation problems for properties that scale nonlinearly is still lacking. Here, more insight is needed, which could be obtained using synthetic studies mimicking real-world conditions as closely as possible or using real-world studies with sound verification. A second multiscale DA problem is when measurements are available at multiple scales. More experience is required with the assimilation of measurements at different scales. In theory, this is easy for problems that scale linearly, but in practice the data could conflict. Studies should not focus solely on the optimal fusion of data, as the bias correction of the observation data is also important [145]. It is also important that the different measurement data be weighted correctly. Expected measurement errors are therefore needed. These are often not well known and require increased ground truth verification. However, the measurement errors of the scaled data sets must be derived from the measurement errors at the original observation scale considering the applied scaling method. If data on two or more spatial scales are to be assimilated, a hierarchical approach constrained by the mass balance or further conditions may outperform a prior fusion. Here, assimilation results may be sensitive to the relative weights attributed to these different data sets, especially when they are observed at different spatial resolutions.

The relative weights are not relevant, if an observation operator is used. Its scaling performance depends on the complexity of the observation operator. Let us take the example outlined in Section 4.1.1, where the antenna weighting of SMOS was used to calculate the magnitude of the update for different pixels. This approach considers additional information about the measurement system. Compared to a statistical scaling, such as calculating the average state of fine-scale data on the coarse scale, it is a more sophisticated approach. A more complex scaling observation operator with respect to the example given is the method published by Merlin *et al*. [196]. In short, SMOS soil moisture on the coarse scale (∼40 km) is downscaled using MODIS soil skin temperature and MODIS NDVI (both fixed to 10 km) amongst others. The MODIS data contain completely new information that is not related to the SMOS soil moisture measurement itself. The method makes use of the finding, that soil evaporative efficiency (estimated based on soil temperature) is directly related to soil moisture.

If the additional data is also a state variable of the main model, they can be assimilated into the model (*i.e.*, multivariate DA) and used in the scaling algorithm as well. As discussed in Section 4.2.1, Merlin *et al*. [135] assimilated this disaggregated soil moisture into a SVAT model. Here, the additional MODIS soil skin temperature can also be predicted by the SVAT model. Therefore, one integrated approach may use the MODIS temperature product to update the simulated soil skin temperature, which can then be used for the observation operator. This may be performed in two steps, first by assimilating coarse-scale soil moisture and fine-scale soil temperature, and then by assimilating downscaled soil moisture in a second step. Another approach may directly insert the fine-scale soil temperature into the disaggregation and assimilate both the fine-scale soil moisture and soil temperature into the SVAT model. The extension of this framework mentioned above refers to MVMS DA, as discussed in Section 6.

### 7.2. Multivariate Single-Scale Data Assimilation (MVSS)

Although many different data types are available to constrain hydrological model parameterization and prediction, relatively few DA papers deal with MVSS DA. In groundwater hydrology, vadose zone hydrology and rainfall-runoff modeling sequential DA is a relatively novel approach with an increasing number of papers only in the last five years. Very few papers are therefore concerned with MVSS DA. The situation is different for land surface hydrology, where sequential DA was first used more than a decade ago. This is also the area with a larger number of papers on multivariate DA. However, it also lacks papers dealing with the assimilation of many different data types. In land surface hydrology, the main topic has been the assimilation of soil moisture data from satellites. This involves serious complications: biased data (e.g., vegetation, interferences), limited vertical penetration depth, nonlinear observation operators and scale mismatch. In addition, land surface hydrological models often use strongly simplified concepts of vegetation and the hydrological cycle, where the assimilation of LAI or FPAR data is not possible (they are a parameter in such models) and the assimilation of river discharge data or groundwater data is difficult because of strong model simplifications.

On the other hand, synthetic experiments have shown that multivariate DA is generally superior to univariate DA. Considering the evolution in the last years, we expect that an increasing number of applications will further investigate the benefits of multivariate DA. We also expect that it will be more successful in real-world applications in the area of land surface hydrology if it is combined with better models (e.g., models that allow lateral flows in the subsurface) and coupled models (e.g., in combination with a crop growth model to assimilate vegetation data). Improving our understanding of remotely sensed data, such as indirect estimates of soil moisture is also essential for increasing the use of MVSS DA (especially in land surface models). In Section 5.1, we showed that a key element in MVSS DA is the relative weighting of the different data types. Therefore, the uncertainty of the measured data must be better understood, and increased ground truth verification is required under different conditions. The equations in Section 5.1 also showed that the impact of the data is greater if measurement errors are smaller. Again, especially for remotely sensed data, a better understanding of the relation between what is measured (e.g., brightness temperature) and what we want to know (*i.e.*, soil moisture) is essential. To conclude, the implementation of MVSS DA schemes and more synthetic test studies are not enough to improve the predictive capacities of terrestrial models. MVSS DA should be combined with improved model structures, an increased understanding of what is being measured and the specification of the relative uncertainty of different measurement data under different environmental conditions.

### 7.3. Multivariate Multiscale Data Assimilation (MVMS)

Multivariate multiscale DA is the most complicated form of DA. For this type of assimilation, both aspects mentioned for multiscale DA (UVMS) and multivariate DA (MVSS) also hold here. Uncertainty assessment and bias correction of measurement data and appropriate multiscale methods to handle nonlinear scaling states and/or parameters are all important aspects. Although the combination of complications makes MVMS DA more problematic, we do not believe that it involves additional theoretical complications compared to MVSS or UVMS DA alone.

To date, only simple aggregation or disaggregation methods have been used to match the spatial resolution between observations and model states. More advanced downscaling methods, such as the downscaling of coarse passive soil moisture products with high-resolution remote sensing data [197,198], have not yet been used in MVMS DA. These methods would be expected to improve the DA performance further. Several publications were concerned with snow DA. The remote sensing products of soil moisture and surface temperature are also diverse in scale and source, and more work is necessary to study the impacts of MVMS assimilation of soil moisture and surface temperature at different spatial resolutions.

### 7.4. Other Aspects

A complication of multivariate and/or multiscale DA where parameters are also updated is that the parameter estimates cannot be directly verified in the field. Therefore, only indirect verification of calibrated parameter values is possible. This can be done by comparing uncalibrated and calibrated parameter values in independent model prediction experiments. If errors are significantly smaller for runs with calibrated parameter values (compared to uncalibrated values), then this would indicate that parameter estimation helped to improve model parameterization. Nevertheless, it is also possible that improved predictions with calibrated parameters are related to the fact that the updated parameter values compensate for another model structural error.

Running high-resolution models with many unknown states (and parameters) in an ensemble mode is very CPU-intensive and large amounts of stored data need to be managed. Small ensemble sizes give suboptimal results and therefore a certain minimum ensemble size is needed. High-performance computing is therefore an essential part of the DA methodology, and this is even more so the case for MVMS DA. For some very CPU-intensive applications, such as land surface modeling, this may have prohibited to some extent a more widespread use of these techniques until now.

## 8. Conclusions

In the context of climate change, several activities have been established for the long-term monitoring of environmental conditions. In general, *in situ* sensors used work in an automated way, and the data is collected in databases, which are often available to the general public with online access. Additionally, the initiation of various national and international space and science programs has ensured a further operationalization of the space infrastructure to provide information on important environmental variables. Available time series of environmental data (also in near-real time) are not fully utilized in terrestrial models. DA is the ideal technique to combine model simulations with observations because it considers the individual uncertainties. Several examples have already been published, e.g., for model calibration, model enhancement, and parameter estimation. The rising complexity of models, observation operators, and measurement systems favors the assimilation of data on different spatial scales as well as different state variables.

In this paper, we reviewed the state of the art of DA utilizing observational data from different spatial scales and different sources. We summarized three prominent DA methods: the ensemble Kalman filter (EnKF), the particle filter (PF) and variational methods (VAR). We identified four major classes of assimilation studies:

- Univariate single-scale DA (UVSS, not discussed here as review papers exist in the various fields of research).
- Univariate multiscale DA (UVMS). This refers to the assimilation of external data obtained at a different resolution than the model resolution. Examples are the assimilation of coarse-scale soil moisture contents or snow water equivalents into a hydrologic model, which is applied at a fine spatial scale.
- Multivariate single-scale DA (MVSS). This refers to the assimilation of data for multiple variables (for example, surface temperature and soil moisture contents) into a simulation model.
- Multivariate multiscale DA (MVMS). This refers to a complex combination of UVMS and MVSS.

We discussed several studies aiming to assimilate observations into models at dissimilar spatial scales. Some applications used the observation operator to align the spatial reference, others used prior downscaling before assimilation. If more than one observation of the same variable was used with a different spatial resolution, the majority of studies fused these data first. In a second step, one combined data set was usually assimilated. This approach may lead to underrepresentation of the variability as well as the accuracy of the state variable.

MVSS DA can be handled by EnKF, PF, VAR and other DA techniques. A crucial role is played by the appropriate determination of the measurement error variances for the different information. MVSS DA has been applied in several studies, but there are still few applications using real-world data or several (more than two) types of data. Most studies concluded that the results obtained when different types of data are assimilated are better than UVSS DA. Examples of this include the joint assimilation of soil moisture and LAI, the joint assimilation of river discharge and soil moisture, or the joint assimilation of surface temperature and brightness temperature. We argued that model deficiencies and an incomplete understanding of the relation between the measurement and the variable of interest has hampered a more extensive use of multivariate DA in the past. In order to ensure the successful application of multivariate DA in the future, these points and an increased understanding of the magnitude of measurement errors under different environmental conditions are important.

In conclusion, methods already exist for the simultaneous assimilation of various data types on different spatial scales. In atmospheric science, multivariate and multiscale DA is well established. In terrestrial systems, they are not yet generally established, and published studies are often synthetic. Further activities are needed to fully exploit the availability of environmental data, which could improve our knowledge of terrestrial processes as well as their interdependencies and teleconnections with the climate system.
